# Dr. Martin K. Bridges

**Published:** 1855-01

**Authors:** 


Obituary.—We are pained to record the death of another of the pioneers of our profession; that of Dr. Martin K. Bridges, who died in Brooklyn, N. Y., on the —— day of October last, aged 54.

Dr. Bridges was born in Hardwick, Mass. For the last twenty years he has been a resident of Brooklyn, and at one time was the only practicing dentist in that city.

For the last two years his health has been gradually declining, owing to a severe attack of paralysis. Since July last, he has rapidly declined, in consequence of the disease terminating in the head, resulting in softening of the brain, from which he died.

Dr. B. was devoted to, liberal and successful in, his profession, and the instructor of many, who now regard him with that gratitude, respect and high appreciation of his many virtues, which could only arise from constant intercourse with him.

Justly esteemed for his kindness of heart, the overflowing benevolence of his nature, which in his better days, found constant expression in good deeds to the suffering and poor, he has gone, we doubt not, to reap the reward of a christian's inheritance.

